# FIGG: Simulating populations of whole genome sequences for heterogeneous data analyses

**DOI:** 10.1186/1471-2105-15-149

**Published:** 2014-05-19

**Authors:** Sarah Killcoyne, Antonio del Sol

**Affiliations:** 1 Luxembourg Centre for Systems Biomedicine (LCSB), University of Luxembourg, Campus Belval, 7, avenue des Hauts fourneaux, Esch/Alzette L-4362, Luxembourg

**Keywords:** Genome sequence, Simulation, Variation frequency, Population

## Abstract

**Background:**

High-throughput sequencing has become one of the primary tools for investigation of the molecular basis of disease. The increasing use of sequencing in investigations that aim to understand both individuals and populations is challenging our ability to develop analysis tools that scale with the data. This issue is of particular concern in studies that exhibit a wide degree of heterogeneity or deviation from the standard reference genome. The advent of population scale sequencing studies requires analysis tools that are developed and tested against matching quantities of heterogeneous data.

**Results:**

We developed a large-scale whole genome simulation tool, FIGG, which generates large numbers of whole genomes with known sequence characteristics based on direct sampling of experimentally known or theorized variations. For normal variations we used publicly available data to determine the frequency of different mutation classes across the genome. FIGG then uses this information as a background to generate new sequences from a parent sequence with matching frequencies, but different actual mutations. The background can be normal variations, known disease variations, or a theoretical frequency distribution of variations.

**Conclusion:**

In order to enable the creation of large numbers of genomes, FIGG generates simulated sequences from known genomic variation and iteratively mutates each genome separately. The result is multiple whole genome sequences with unique variations that can primarily be used to provide different reference genomes, model heterogeneous populations, and can offer a standard test environment for new analysis algorithms or bioinformatics tools.

## Background

This paper introduces the FIGG (Frequency-based Insilico Genome Generator) tool, which is designed to be of use to computational researchers who require high volumes of artificially generated genomes that mimic the variation seen in the natural population. FIGG is designed to use high performance computing to rapidly generate artificial genomes, and can be used to generate large numbers of similar whole genome sequences by iteratively seeding each run with new parent genomes.

In the last few years high-throughput sequencing (HTS) has allowed researchers to sequence genomes for species that range from bacteria and plants, to insects and vertebrates. In the context of biomedicine HTS is being used to: characterize complex ecologies such as the human gut microbiome [1]; understand parasitic diseases such as malaria [2]; identify genomic variations that may be responsible for virulence in diseases such as tuberculosis [3]; and search for the mutations that drive genomic diseases such as cancer [4-6].

A result of this wide-ranging use of sequence information is petabytes worth of genomic data across multiple species, populations and diseases. New tools are constantly being required to enable the management and analysis of this information. The FIGG tool is meant to be of use to different computational researchers working in the area of large-scale genomics. In particular it is designed to be used by those who are struggling to keep pace with the scale and diversity of data in large-scale genomic projects. Using FIGG to generate artificial data has a number of advantages over downloading and storing publically available whole genome sequences as it: has known characteristics, so can be used for consistent benchmarking; can be used to generate mixed populations of heterogeneous genomes for algorithm testing; has no security requirements, so can be shared and used more easily; and does not place undue load on local resources, as genomes can be generated on the fly.

FIGG is designed to generate large volumes of potentially related sequences that can be used by computational researchers in testing their models, analysis pipelines and informatics solutions. Simulating experimental data is a common step in the development and evaluation of new analysis tools [7], computational methods, and the support infrastructure for managing such sequences. Many different genomic simulators are available (see Table 1) and have been described elsewhere [8], however these are not designed to provide the high volumes of complete genome sequences which are required for software testing and algorithm development. They range in application from instrument-specific sequence read simulation (e.g. ART [9], MetaSIM [10]), to genotype simulation for case–control studies based on linkage disequilibrium patterns (e.g. genomeSIMLA [11], GWASimulator [12]), to evaluating a population over time to determine how genomic hotspots or population bottlenecks affect a genome (e.g. FreGene [13], GENOME [14]) or protein sequence (e.g. ALF [15]).

**Table 1:** Genome simulators

**Tool**	**Description**	**Outputs**
ART [9]	Simulation of sequence reads with error models for multiple platforms (454, Solexa, SOLiD).	Single or pair ended sequence reads.
MetaSIM [10]	Simulation of sequence reads for metagenomics, particularly for highly variable data (taxonomically distinct but related organisms).	Single or pair ended sequence reads.
GENOME [14]	Population simulation within a set of alleles using genome level events such as recombination, migration, bottlenecks, and expansions.	Alleles identified as mutated (1) or not (0) across the simulated population.
GWASimulator [12]	Simulation of loci across a population which follows a given LD structure in case–control type studies.	SNVs per individual for input loci.
FreGene [13]	Mutation simulation using a theoretical sequence of a given size with hotspot, conversion, and selection parameters.	Mutation selection across population for a theoretical sequence.
genomeSIMLA [11]	Simulation of disease loci within a family or case–control setting using specific LD patterns for investigations of disease.	Affy identified SNPs selected by disease association.
ALF [15]	Population simulation for a specific gene set using a model for variation at the sequence and individual level.	FASTA protein and DNA sequences for specific genes.

Example simulators used in various types of genome investigations. Many use the Wright-Fisher model of population genetics theory [8] in order to generate populations that vary over time given some set of event frequencies such as LD, hotpots, population bottlenecks (GENOME, genomeSIMLA, FreGene), others provide a set of sequences that could be generated by a given sequencing technology with an error model (ART and MetaSIM). The specific simulator used is based on the type of investigation. In planning new GWAS studies for instance, a simulator that uses LD patterns and can provide predicted genomic regions for disease related mutations would be selected. However, such a simulator would not be of use in the planning of a metagenomic study for an organism which may not yet be fully sequenced, or is highly variable. None of these simulators provides whole genome FASTA as outputs.

FIGG generates whole genome sequence files, in FASTA format, by directly sampling from populations of observed variations. Each artificial genome includes sequence mutations that range from single nucleotide variations (SNV) to small and large-scale structural variations (e.g. indels, tandem duplications, inversions). It has been designed to use a distributed computing framework to enable rapid generation of large numbers of genomes while tracking the mutations that are applied to each. Below we provide details of the FIGG methods that enable the creation of diverse whole genomes which accurately model experimentally derived real sequence data. The following sections describe the methods used for analysis of background genomic variation, generation of the sequences, and validation of the models through the use of standard sequence analysis tools. Finally we discuss applications for FIGG within the sequencing community.

## Methods

FIGG requires two inputs in order to create a genome: 1) all FASTA files representing the chromosomes to be simulated (e.g. chromosomes 1–22, X, and Y from human genome build GRCh37), and 2) a database that is the result of the frequency analysis as described in the next section (the full database format can be found at the link provided in Availability). The resulting output from FIGG is set of FASTA formatted sequence files (one per chromosome) that can be used by any tools which use FASTA as an input, including sequence-read simulators and genome alignment software.

### Variation frequency analysis

The public availability of large datasets that characterize human genomic variability provide a wealth of data on population and individual variations. In order to develop an accurate estimate of the range of "normal" variation we used Ensembl [16]. This data was mined for all variants validated in the 1000Genomes [17] and HapMap [18] projects, as these are generally considered representative of normal populations. Several other sources representing disease variations were downloaded for comparison, including those from the Catalogue of Somatic Mutations in Cancer (COSMIC) [19] and small structural variants in the Database of Genomic Variants Archive (DGVa) [20].

**Figure 1:**
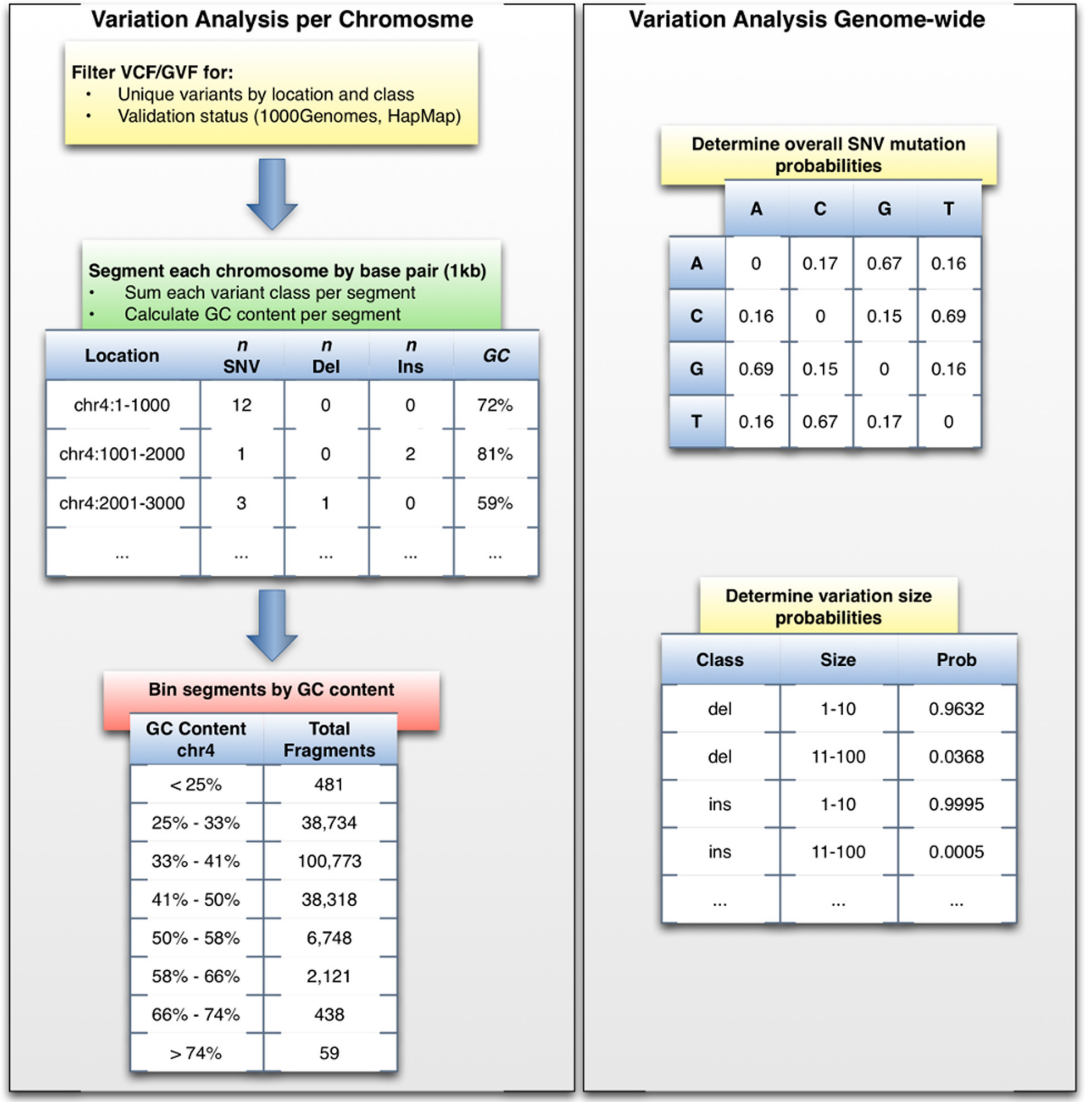
**Variation frequency table generation procedure.** The variation analysis uses publicly available small scale variation data to generate a set of database tables for a specific variation frequency. This is done in four separate steps. First, filter GVF or VCF files for unique variations per chromosome location and validation status. In this analysis variation files from Ensembl were used and "normal" validation status was determined based 1000Genomes or HapMap annotations. To generate a "highly variant" frequency, variations that were identified as being in the COSMIC and DGVa databases were added. Next, each chromosome is segmented into defined lengths (e.g. 1 kb) and the observed variations per class within the segment are counted. Additionally, the GC content for each segment is calculated from a corresponding FASTA sequence file. Then the segments are separated by GC content into 10 bins per chromosome. While these bins can be more granular, the correlation of SNV to GC content did not improve by increasing the number of bins. Finally, determine the genome-wide SNV mutation and size probabilities for variations that can be more than a single base pair in length. A database schema describing the final tables is provided in the source for FIGG.

In order to characterize the variant frequency across the genome for different classes of mutations each chromosome was first fragmented into base-pair lengths that were manageable for processing. For each fragment a profile of unique variants was developed. These profiles were then analyzed to determine the frequency of each variant class: single point mutations being the most common, followed by sequence alterations (defined as an uncharacterized change in the sequence), and then insertions. Based on these frequencies structural elements in the sequence fragment were identified that can be directly observed and which could explain the variation frequencies including: a higher incidence of coding/non-coding regions; predicted CpG methylation sites; and high/low GC content. A weak correlation with SNVs was observed in segments with high/low GC content [21,22], but no other genome-wide structural correlation was found. When the same analysis on "disease" variations was run (e.g. COSMIC, DGVa) as a comparison, GC content continued to be the only clear structural correlation for variation frequency (see Figure 1 for a description of the final output).

**Figure 2:**
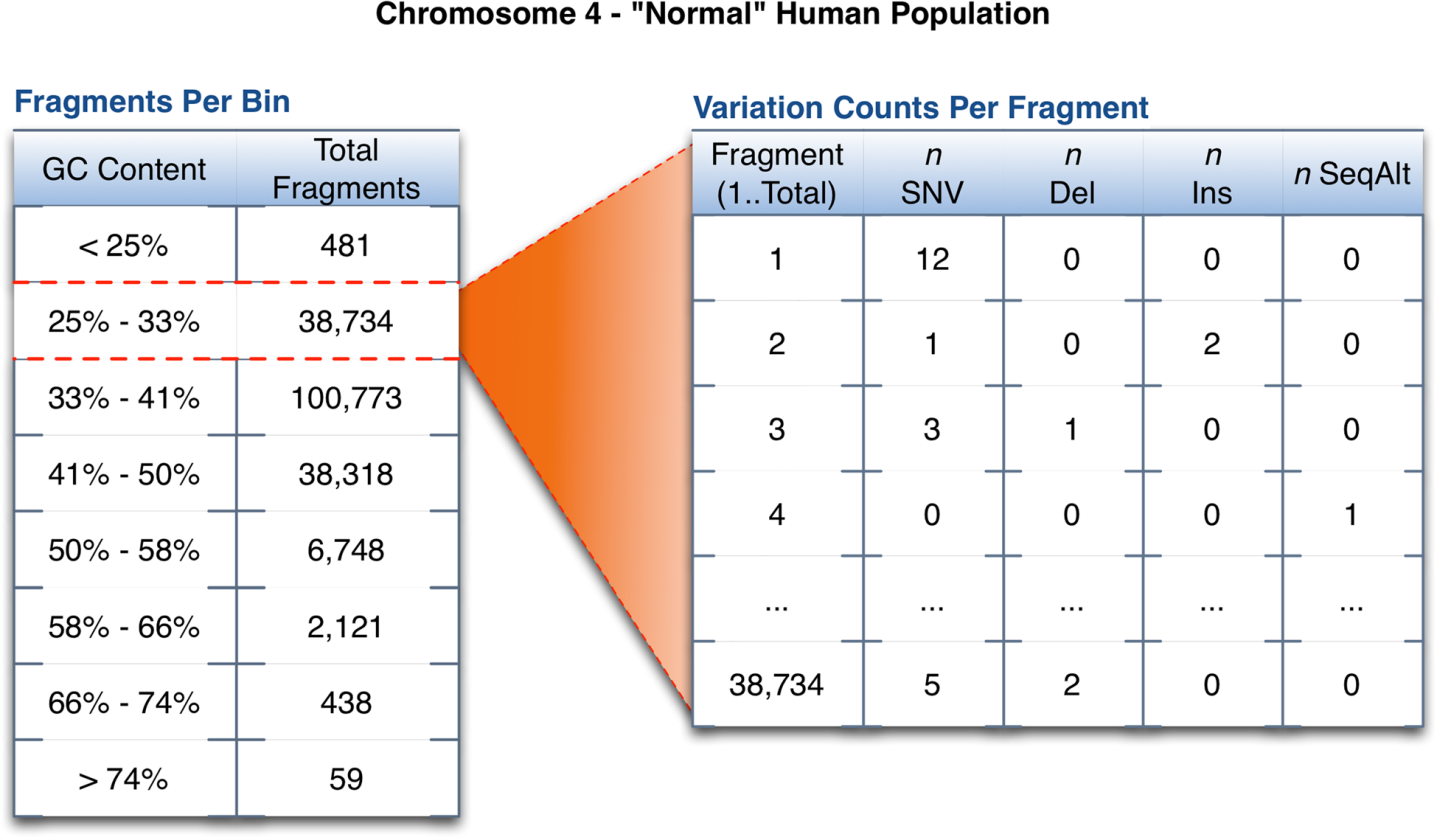
**Variation frequency analysis.** The result of the variation analysis is a table, indexed by chromosome and GC content, which provides experimentally observed counts of the different variations for that fragment. This means that a DNA fragment from chromosome 4 with a GC content of 25-35% has been observed 38,734 times. Each of those observed fragments is recorded with their variant counts. These observed fragments will be sampled from directly in the generation of an artificial genome.

Based on this analysis the observed sequence fragments were separated into bins by GC content, with variant counts per segment recorded for each chromosome (see Figure 2 for an example of the variant and GC tables in chromosome 4). The result is a set of tables that can be easily sampled for fragments based on a GC profile. Additionally, base pair size probabilities were calculated for all size-dependent variants (e.g. deletion sizes from 1–10 have a genome-wide frequency of 0.96, and from 11–100 a frequency of 0.04), and nucleotide mutation rates were determined for SNVs (e.g. C- > T 0.69, C- > A 0.16, C- > G 0.15, etc.).

## Implementation

**Figure 3:**
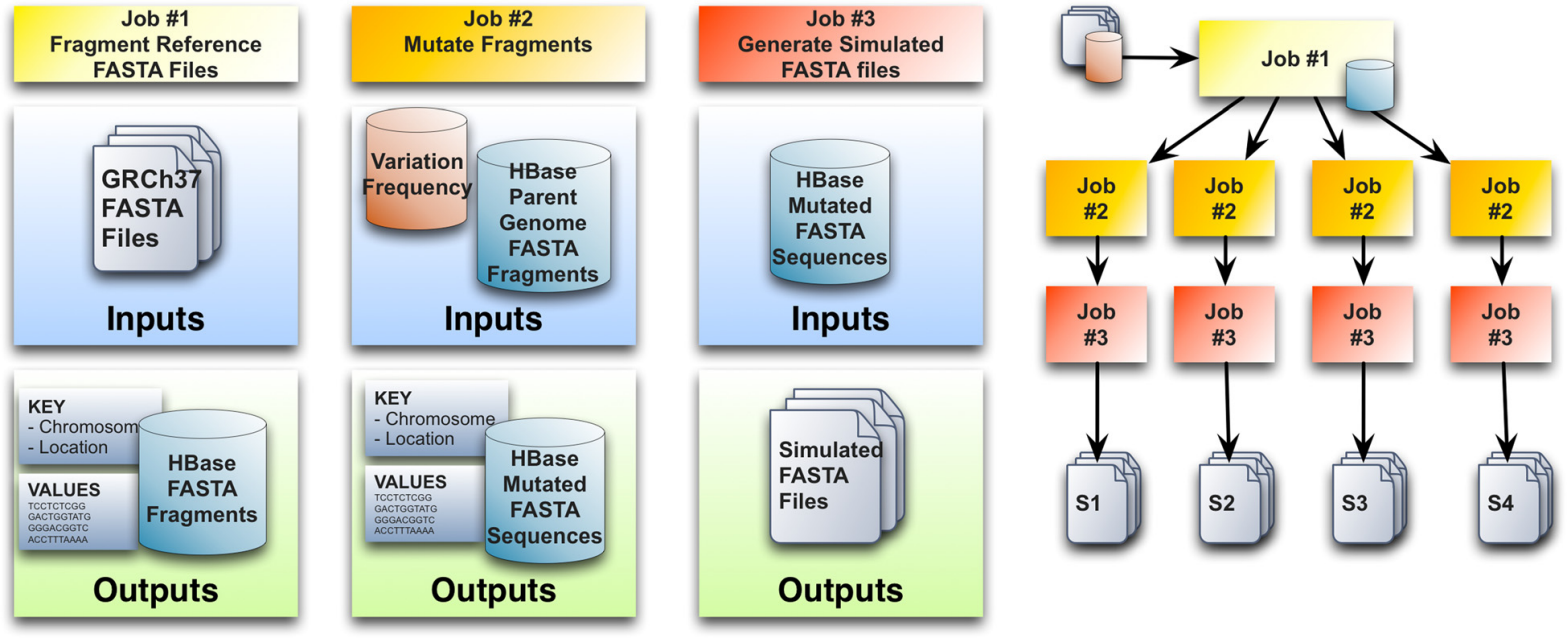
**FIGG MapReduce jobs.** Three discrete MapReduce jobs have been set up to generate unique whole genome sequences. The first job simply fragments the reference or "parent" genome into the distributed database, HBase. The second job reads all the fragments for the parent genome from the database, mutates them using the provided frequency information and again saves them to the database to ensure reproducibility. The final job generates FASTA formatted files, per chromosome, for the mutated genomes.

The general architecture of FIGG is shown in Figure 3. It has been designed to take advantage of distributed computing by both breaking down the processing of the data into a distributed model, and by separating the functionality required into distinct steps, called "jobs", that can be added or altered for downstream analysis or testing needs. FIGG is separated into three distinct jobs. The Additional file 1 document provided describes how to set up and run these jobs on an Amazon Web Services cluster.

The first job fragments a reference genome and persists it to a distributed database, which ensures that the background genomic information is highly accessible, and only needs to be run once per reference (e.g. GRCh37).

The second job mutates each of the segments from a parent genome, using information pulled from a variation frequency database. This database provides the information necessary to determine which variations should be applied to a given fragment (e.g. SNV, deletion, insertion) and how often these occur.

The third job assembles the mutated fragments into a whole genome, and generates the corresponding FASTA files. The second and third jobs are run in parallel to each other, allowing for a means to generate large numbers of artificial genomes in a highly scalable manner.

### Mutation rules

**Figure 4:**
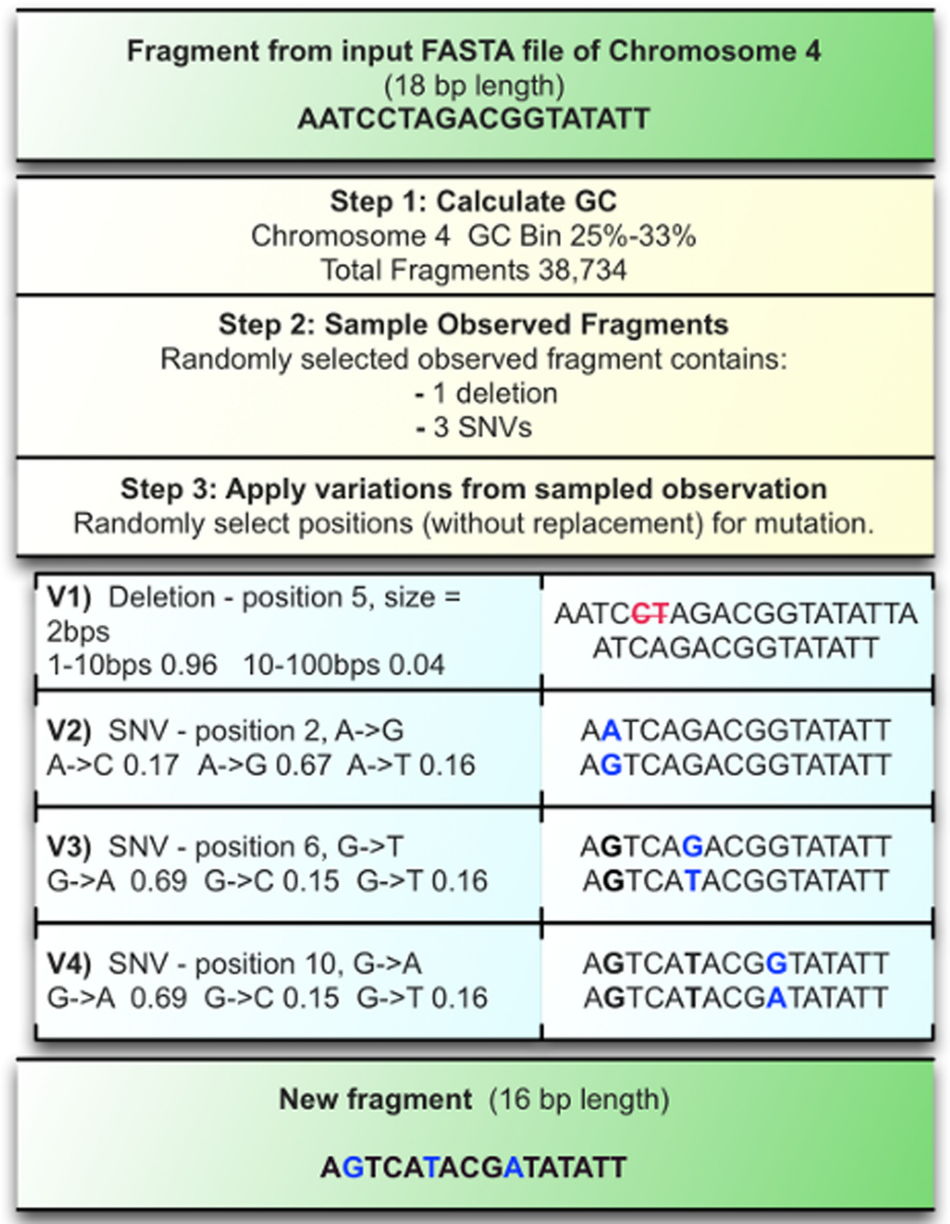
**Fragment mutation rules.** As an example of the process each fragment goes through, this fragment from chromosome 4 is mutated based on information from the tables shown in Figure 2. In step 1 the GC content of the fragment is calculated then fit to the pre-determined bins, all observed fragments within that bin are then available to sample. Step 2 samples one of these observed fragments to get the counts of specific variants. In this case the observed fragment had a single deletion and three SNVs. In step 3 these observed variant counts are applied in stages. Sites for each variation are selected randomly (without replacement), and the mutation applied. For a size-dependent variant such as the deletion, a size is determined from a probability table, for SNVs the probability of the point mutation is determined based on the nucleotide present at that site. The resulting fragment will not replicate the sampled fragment (from step 2) in specific mutations, but only in the number of mutations applied.

The generation of new, mutated sequences is achieved through application of a ruleset based on the frequency analysis described above. Each input chromosome is split into fragments of the same size as those used for the frequency analysis (e.g. 1 kb). Each fragment is then processed stepwise (see Figure 4):

1. Determine the GC content of the fragment then fit to the identified bins in the frequency database based on the fragment chromosome. This provides a set of observed fragments to sample.

2. Randomly sample an observed fragment from the set of fragments that fit the GC bin. This fragment will include 0..*n* counts for each variation type (e.g. SNV, deletion, substitution, etc.).

3. Apply each variant type to the fragment sequentially (e.g. deletions first, tandem duplications last). This is achieved through sampling without replacement random sites within the fragment for each mutation, applying size-dependent or SNV probabilities for that mutation to the site, and repeating until all variants have been applied to the sequence.

The resulting fragment may vary significantly from, or be nearly identical to, the original sequence depending on the selected variant frequencies. Use of random site selection for applying the mutations ensures that no specific population bias (e.g. if the population that is used to generate the frequency data is overrepresented for a specific variant) is introduced into the bank of resulting sequences. The final FASTA sequence then provides a unique variation profile.

### MapReduce for multiple genomes

Applying this process to the human genome to create a single genome is slow and inefficient on a single machine, even when each chromosome can be processed in parallel. In fact, a basic version of parallelization took more than 36 hours to produce a single genome. Producing banks of such genomes this way is therefore computationally limited. However, mutating the genome in independent fragments makes this a good use case for highly distributed software frameworks such as Apache Hadoop MapReduce [23,24] backed by distributed file systems to create and store tens, hundreds, or more, of simulated genomes. In addition, use of HBase [25] allows for highly distributed column-based storage of generated sequences and mutations. This enables rapid scale-up for management, ensures that all variations to a given genome can be identified, and allows for the simple regeneration of simulated FASTA files on an as-needed basis.

MapReduce has been used effectively by us and others in various large-scale genomics toolsets to decrease computation times, and increase the scale of data that can be processed [26-28]. FIGG uses this framework in order to allow the rapid generation of new genomes or regeneration of previous mutation models. It is designed to run in three discrete jobs: 1) breakdown input FASTA files into fragments and save to a HBase database for use in subsequent jobs; 2) mutate all of the fragments from the first job and persist these to HBase; and 3) reassemble all mutated fragments as new FASTA formatted sequences.

**Figure 5:**
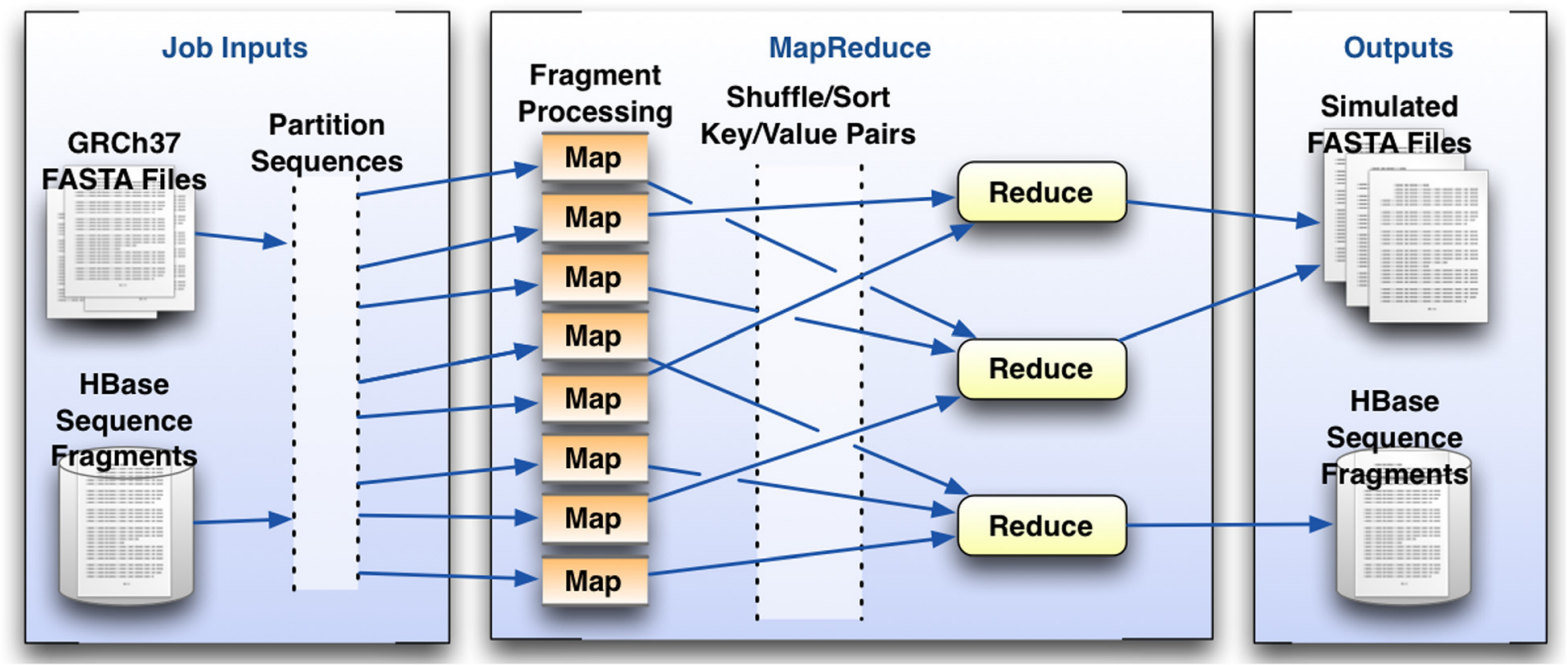
**MapReduce framework.** MapReduce provides a general framework to process partitionable data. The Map phase may either gather metadata statistics on a sequence fragment and write them to HBase (Job 1) or apply the variation frequencies and rules to a fragment (Job 2). The Reduce phase, if it is specified, is responsible for assembling the mutated fragments into FASTA formatted chromosome files (Job 3) or it may simply output additional metadata to HBase for use in other processing tasks.

MapReduce accomplishes these tasks by breaking each job into two separate computational phases (see Figure 5). The *Map* phase partitions data into discrete chunks and sends this to mappers, which process the data in parallel and emits key-value pairs. In each of the separate jobs for FIGG the mappers deal with FASTA sequences, either directly from a FASTA file or from HBase. Each mapper performs a computation on these sequences, and produces a sequence (the value) with a key that provides information about that sequence (e.g. chromosome location). These key-value pairs are "shuffle-sorted" and picked up by the *Reduce* phase. The framework guarantees that a single reducer will handle all values for a given key and that the values will be ordered.

It is worth noting that not all jobs will require the use of a reducer. In FIGG the first job which breaks down FASTA files into fragments and saves them to HBase (Job 1) is a "map-only" job, because we cannot further reduce these fragments without losing the data they represent. Therefore, the mappers output directly to HBase rather than to the reducers. In the mutation job (Job 2) the *Map* phase performs multiple tasks including applying variations to a sequence fragment, and writing new sequences and specific variation information directly to HBase. Whereas in Job 3 (FASTA file generation), the *Map* phase only does a single task, tagging a sequence with metadata that enables it to be ordered for the *Reduce* phase, which actually outputs the file. As each mapper is processing a subset of the data in parallel to all other mappers the compute time required will scale directly with the number of mappers available to the task, limited in FIGGs case only to the organization of the data in HBase.

## Results and discussion

Our primary interest in developing this tool was to provide sets of heterogeneous whole genomes in order to benchmark cancer genome alignments. This is a special case for alignment, as cancer genomes can vary quite dramatically between patients and even within a single tumor. With such a range of variation in patients, it was important to ensure that the simulated genomes were representative of the heterogeneity, without introducing biases for specific mutations.

In order to ensure that FIGG was modeling heterogeneous genomes that fit a specific background (e.g. "normal" or "diseased") two different frequency backgrounds were generated (see Methods). The "normal" frequency background was from data representative of the average human population: 1000Genomes and HapMap. The second, "highly variant" frequency background was based on data from the DGVa and COSMIC databases of cancer and other disease variations. This greatly increased the frequency and size of the small structural variations (e.g. millions of small deletions and insertions, up to several hundred bp in length).

Using these two different backgrounds and GRCh37 as the parent genome, FIGG generated six whole genome sequences: three "normal", two "highly variant", and one additional genome from the "normal" background that included a common cancer structural variation. As expected, for both the "normal" and the "highly variant" sequences, the simulated genomes preserved the frequency distribution of variations observed in the background data, while differing in the raw counts per fragment.

These simulated whole genomes were then used as references to align a set of low-coverage paired-end sequencing reads from the 1000Genomes project (NCBI Trace Archive accession ERX000272). The BWA alignment tool [29] was used to index the simulated genomes and align the reads against each reference, including the current reference genome GRCh37. Statistics regarding read mapping accuracy (see Table 2) for each genome were generated using SAMtools [30].

**Table 2:** Sequence alignment statistics for simulated genomes

	**SAMtools flagstat**		
	***Mapped***	***Correctly paired***	***Singletons***
**GRCh37**	**98.22%**	**96.34%**	**0.85%**
**S1**	97.89%	95.52%	1.00%
**S2**	95.46%	92.95%	1.09%
**S3**	97.89%	95.54%	0.99%
**S4H**	90.09%	85.11%	2.89%
**S5H**	90.35%	85.45%	2.84%
**S6SV**	88.16%	83.22%	2.88%

A comparison of the 1000Genomes reads for ERX000272 mapped against each genome. GRCh37 is the current reference genome. S1, S2 and S3 are genomes generated based on normal variation data. S4H and S5H were generated with high variation data and S6SV is based on normal variations but with the chromosome arm 19q deleted. The table columns are statistics provided by SAMtools flagstat: *Mapped* provides the total percentage of reads that mapped to the genome on the left; *Correctly Paired* provides the percentage of reads that aligned to the genome in their proper pair; and *Singletons* provides the percentage of reads that were orphaned in the alignment. As expected, genomes S1-3 show mapping statistics that are close to the reference genome, while the others show a significantly lower statistics due to the higher frequency and larger bp size of variations used to generate these genomes.

This comparison demonstrates that heterogeneous a whole genome sequences matching specific variation characteristics (e.g. normal, disease variant, etc.) can be generated by this tool. In the first three genomes the characteristics come from a "normal" population frequency and fairly closely match the mapping rates of the current public reference (GRCh37). The lower mapping rates in the high variation genomes are expected, as these will have a higher number of variations as well as longer insertions, deletions, and substitutions. This suggests that by using distributions for variations within distinct genomic populations, such as can be seen in different tumor types, highly specific simulated genomes can be generated. These specific simulated genomes could then be used as more accurate quality control sets for testing hypotheses or data. For instance, genome S6SV models a breakpoint that may be found in specific types of glioma [31-33]. This simulation could therefore be used to more accurately align a clinically derived sequence, integrate with proteomics data to infer a potential effect or biomarker, or simply provide a test sequence for breakpoint analysis methods [34].

**Figure 6:**
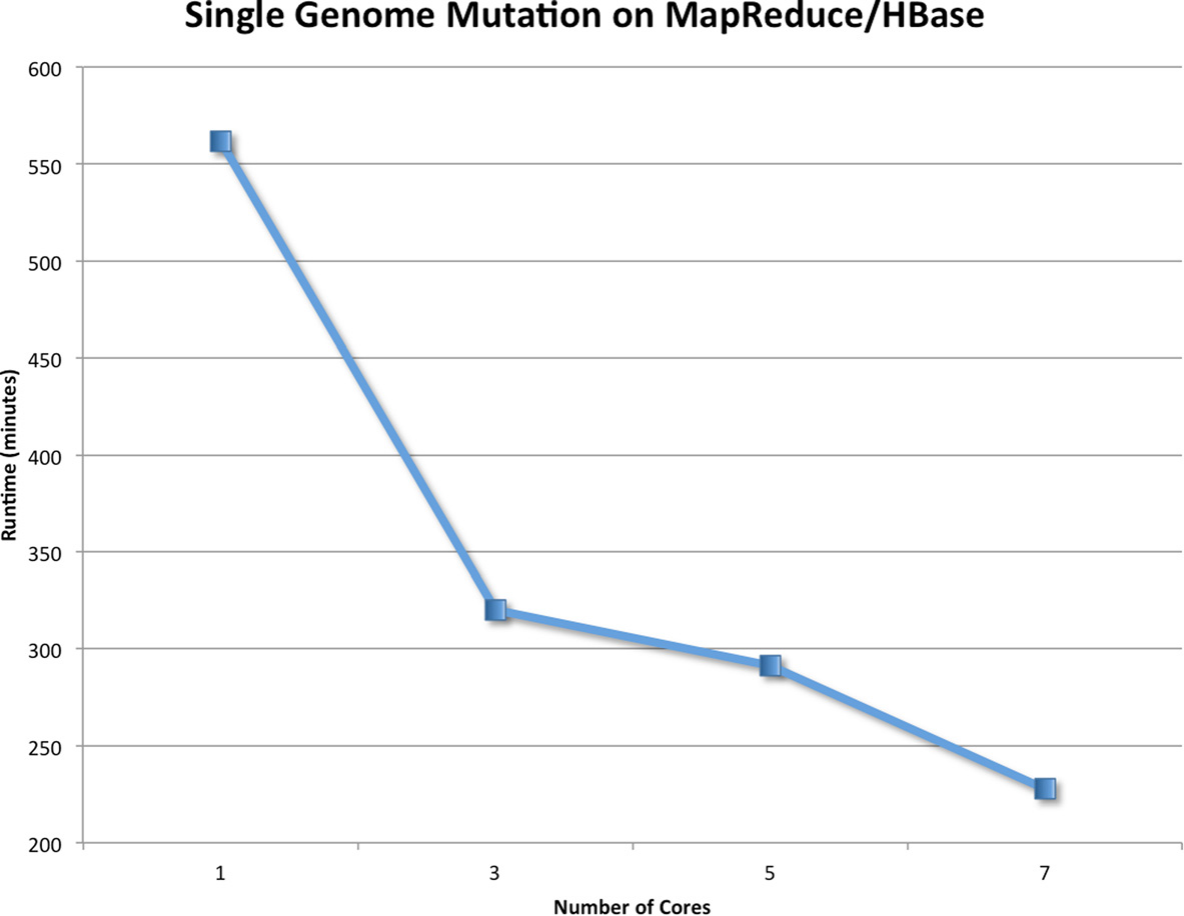
**Scaling FIGG with MapReduce.** The mutation process in FIGG is the most computationally intensive job in the pipeline. It was tested on Amazon Web Services Elastic MapReduce clusters of varying sizes for scalability. MapReduce provides a near linear speed up with the addition of nodes to this job. These genomes are saved to HBase to provide a persistent store of standard artificial genome data that can scale along with the cluster size. This is one area where optimization will provide increased performance as defining how the HBase tables are distributed can increase the speed of computation (e.g. more efficient row key design decreases query time and increases the number of available mappers). This is due to the fact that region server optimization is highly specific to the data, and improves as the data size increases.

Finally, it is important to note the benefits of using a highly distributed framework to generate these sequences. Current sequencing projects are generating hundreds or thousands of sequences from patients. In order to provide artificial data models to assist computational researchers working on large-scale projects, the simulation tool must be able to rapidly generate data of similar complexity and size. Distributed computing frameworks enable FIGG to generate this data quickly, allowing the researcher to simulate the scale of data they will actually be facing. Using Hadoop MapReduce enables FIGG to scale the mutation job nearly linearly to the number of cores available (see Figure 6). However, as with other distributed environments optimization for large clusters must be done on an individual basis.

## Conclusions

HTS is now a primary tool for molecular biologists and biomedical investigations. Identifying how an individual varies from others within a population or how populations vary from each other is central to understanding the molecular basis of a range of diseases from viral and parasitic, to autoimmune and cancer. As our understanding of these variations increases so too does the complexity of the analyses we need to undertake to find meaning in this data.

Simulation data is a common measure of the usability and accuracy of any analysis tools, but in whole genome studies there continues to be a lack of standard whole genome sequence data sets. This is especially problematic with the production of hundreds or thousands sequences from different populations. Comparing these to a single reference can lead to loss of important variation information found in even reasonably homogenous data. Highly heterogeneous populations, such as those found in cancer, may not even be represented at all by the reference. Generating thousands of whole genome models that vary predictably can provide highly specific test data for computational biologists investigating tumor diversity, software engineers who are tasked with supporting the large scale data that is being generated, and bioinformaticians who require reliable standards for developing new sequence analysis tools.

Central to each of these research needs is the development and use of banks of whole genome simulation data which will allow for the development of quality control tools, standard experimental design procedures, and disease specific algorithm research. FIGG provides simulation data models based on observed population information, will enable disease sequence modeling, is designed for large-scale distributed computing, and can rapidly scale up to generate tens, hundreds, or thousands of genomes.

## Availability and requirements

**Project name**: Fragment-based Insilico Genome Generator

**Home page**: http://insilicogenome.sourceforge.net

**Operating systems**: Platform independent

**Language**: Java

**Other requirements**: Java version 1.6 or higher, A computational cluster running Hadoop v1.0.3 and HBase 0.92 (Amazon Web Services AMI v2.4.2), pre-computed HBase tables for the frequency analysis, and FASTA files for a reference genome.

**Open source license**: Apache 2.0

**Restrictions for use**: None

All Hadoop MapReduce jobs for this paper were run using Amazon Web Services MapReduce clusters. Please see the Additional file 1 for a walkthrough of the AWS job creation.

## Abbreviations

COSMIC: Catalogue of Somatic Mutations in Cancer; DGVa: Database of genomic variants archive; HTS: High-throughput sequencing; SNV: Single nucleotide variation.

## Competing interests

The authors declare that they have no competing interests.

## Authors’ contributions

SK and AS conceived of, and planned project. SK analyzed variation data, implemented software and validated results. All authors read and approved the final manuscript.

## Supplementary Material

Additional file 1 Amazon Web Services FIGG Walkthrough. Click here for file
