# Supplementary material for: FIGG: Simulating populations of whole genome sequences for heterogeneous data analyses
Source: BMC Bioinformatics. 2014 May 19;15:149. doi: 10.1186/1471-2105-15-149 (PMC4039316; doi:10.1186/1471-2105-15-149)
Supplement: Additional file 1 — Amazon Web Services FIGG Walkthrough. [file 1471-2105-15-149-S1.docx]

# Supplemental - AWS FIGG Walkthrough

All of the steps described below can be set up on any cluster running Apache Hadoop and HBase. However, as the performance estimates were made using Amazon Web Services (AWS) this walkthrough details how the steps are set up through that service. This walkthrough presumes that the user has an account with Amazon and has signed up for the Elastic Compute Cloud (EC2), Simple Storage Service (S3), and Elastic MapReduce. For detailed information regarding AWS cluster configuration please see the AWS documentation. (<https://docs.aws.amazon.com/ElasticMapReduce/latest/DeveloperGuide/emr-plan.html>)

### Required Files

Download the HBase-Genome-1.1.jar file from <http://insilicogenome.sourceforge.net> and hbase-tables.tgz file from <http://s3-eu-west-1.amazonaws.com/fragment-mutation/hbase-tables.tgz>. Uncompress hbase-tables in a temporary location on your machine. The following directories contain the sequence fragments and variation frequency data:

- Sequence data (GRCh37): genome, chromosome, sequence, small_mutations
- Variation data: gc_bin, snv_probability, variation_size_probability, variation_per_bin

If necessary, follow the instructions on <http://insilicogenome.sourceforge.net> to generate the fragment database from local FASTA files. The variation frequency tables are also available at that link.

### Setup

1. Create a S3 bucket from the web UI, selecting the appropriate region. This bucket will contain the jar file that Hadoop will use, the required data files which will be loaded into HBase, and the log files that are output by Hadoop.
2. Select your newly created bucket and upload the Hbase-Genomes-1.1.jar file.

|  |
| --- |
| \| 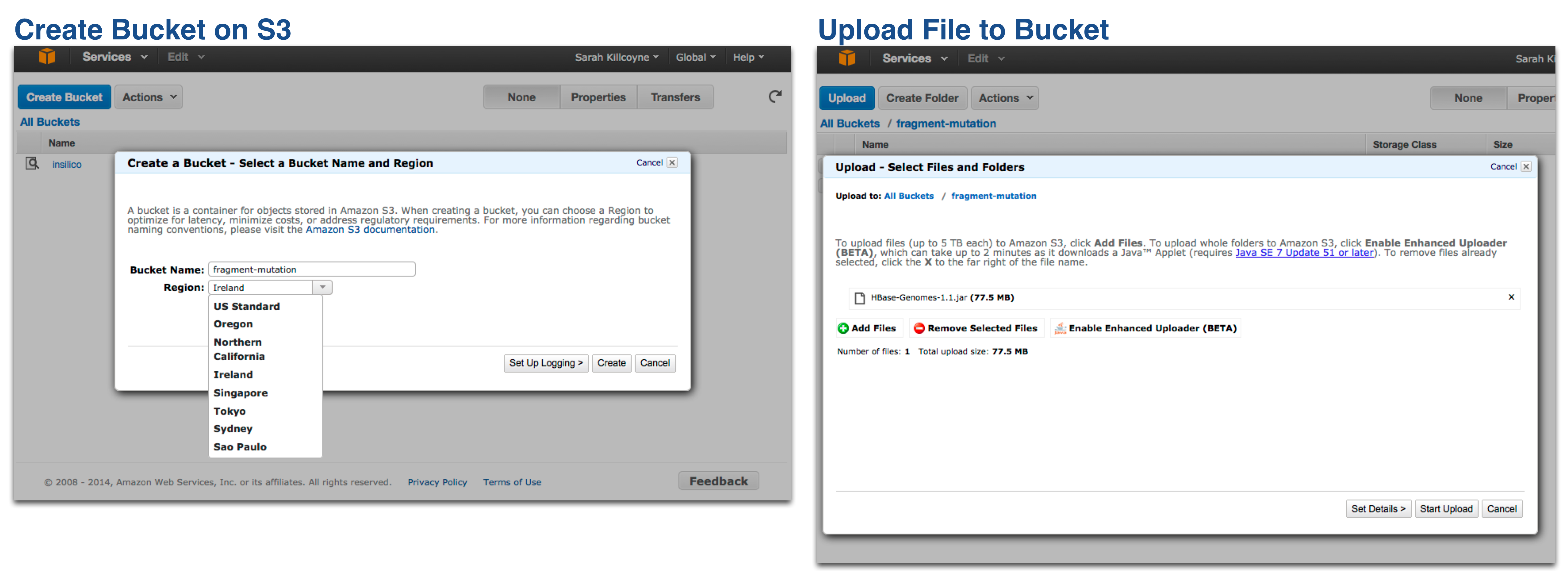 \| \| --- \| \|  \| |

1. Inside your bucket create two folders: one for storing the data files to be loaded into hbase; one for log files from Hadoop.
2. Select your hbase folder and upload all of the directories that were in the uncompressed hbase-tables.tgz file. These must be uploaded as directories, so if you do not use the S3Fox Organizer, you will need to enabled Amazon’s “Enhanced Uploader”.

### Elastic MapReduce Cluster Creation - Web UI

1. Name your cluster and enable logging. Enter the S3 location for your log directory (e.g. s3://mybucket/logs). It is recommended that you disable Termination Protection to ensure that the cluster shuts down when all work is complete. If you do not enable this, you will need to manually terminate the cluster when you are through.
2. Ensure that the AMI version 2.4.2 is selected under “Hadoop Distribution”.

| 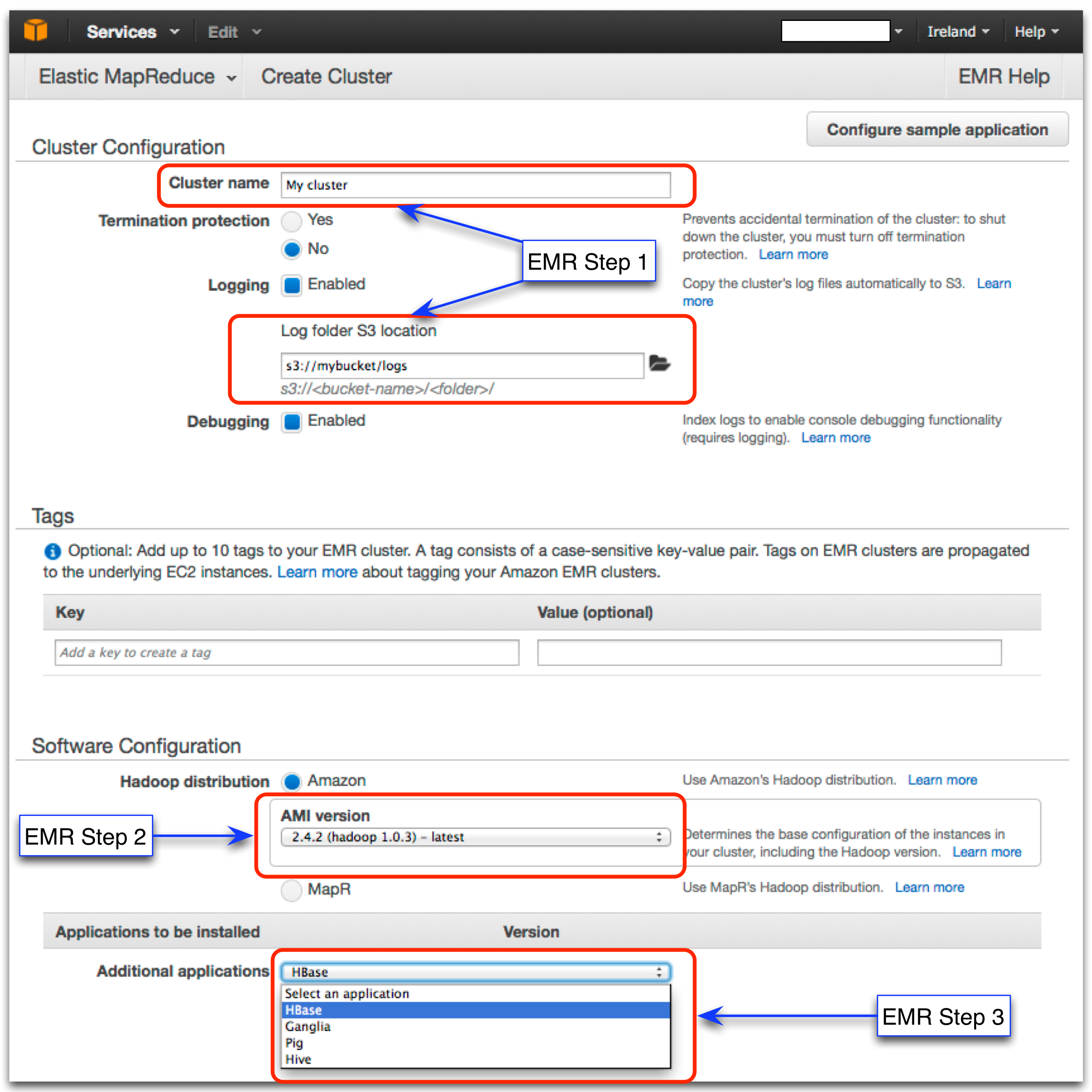 |
| --- |

1. Under “Applications to be installed” remove Pig and Hive (added automatically) and add HBase. No backup or restore needs to be configured.
2. In the next section under “Hardware Configuration” select how many cores you would like to use. A single Master is required. It is recommended for a FIGG run that only Core instances be used, as Tasks are not guaranteed to be available and could result in inconsistent data in HBase.
3. Add a custom bootstrap action to configure HBase. This is important! These configuration options keep the HBase connections live in between tasks:

| The S3 location is s3://<your region>/bootstrap-actions/configure-hbase | |
| --- | --- |
| -s hbase.rpc.timeout=120000 | |
| –s hbase.regionserver.lease.period=120000 | |
| -s hbase.regionserver.handler.count=30 | |
| 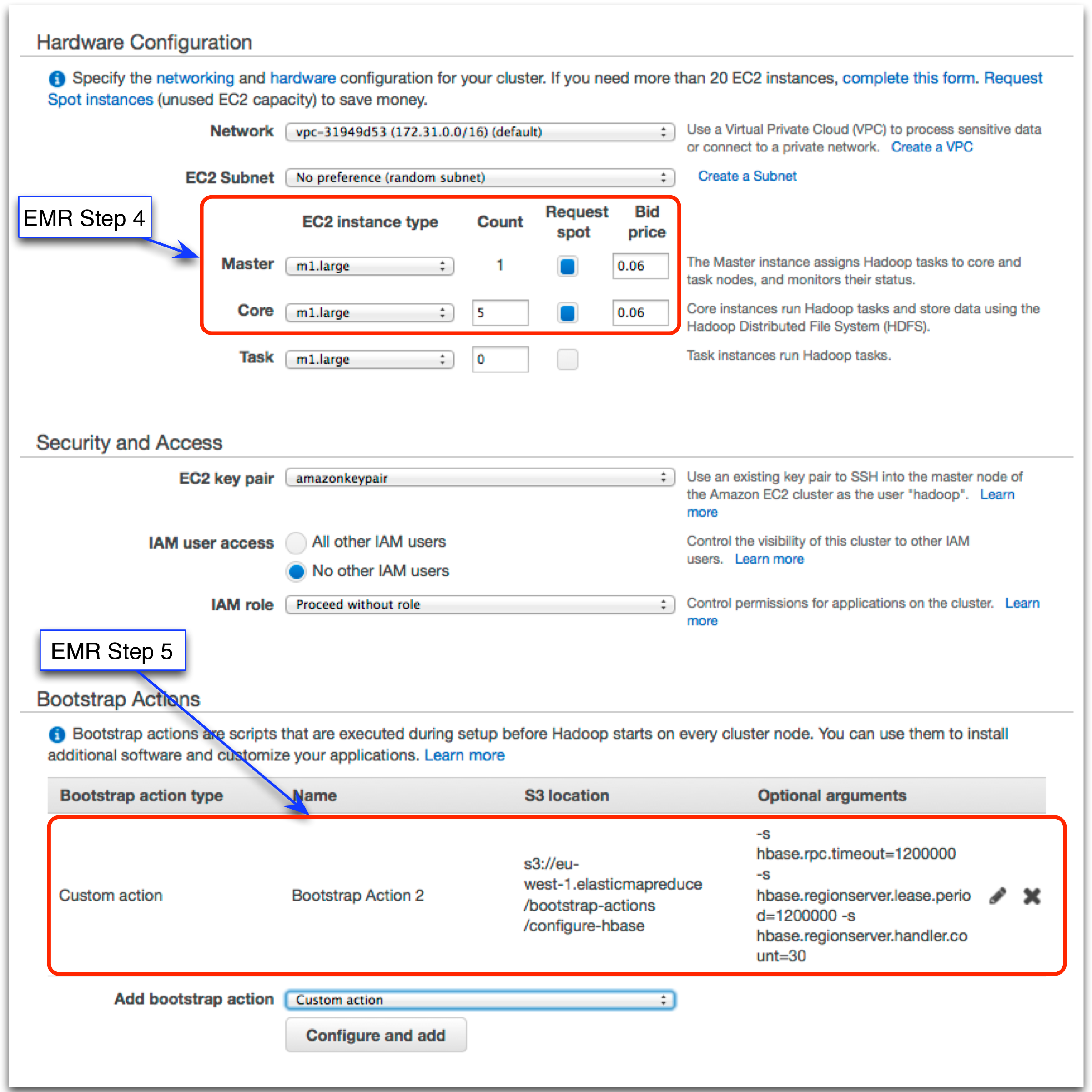 | |

#### MapReduce Steps

Finally, the steps for the actual work that will be performed on the cluster can be specified. Each of these steps is created by selecting the “Custom Jar” in the “Add step” drop down then clicking “Configure and add”. Each step uses the same jar file which you uploaded previously (s3://mybucket/HBase-Genomes-1.1.jar).

| Step Description | Program name | Arguments |
| --- | --- | --- |
| 1. Import Genome Fragments | hbaseutil | -d s3://mybucket/hbase  -c IMPORT  -t genome,chromosome,sequence,  small_mutations |
| 2. Import Variation Tables | hbaseutil | -d s3://mybucket/hbase  -c IMPORT  -t gc_bin,variation_per_bin,snv_probability,  variation_size_probability, |
| 3. Mutate Fragments | mutate | -m MyGenomeName  –p GRCh37 |
| 4. Export Genome Tables | hbaseutil | -d s3://mybucket/hbase  -c IMPORT  -t genome,chromosome,sequence,  small_mutations |
| 5. Generate FASTA files | gennormal | -g MyGenomeName  -o s3://mybucket/myfolder |

These steps will be run in order, and must be set up as shown in the table above. Steps 1 and 2 consist of setting up the data that will be used and must be finished before the mutation can be run. If either of these fail (due to HBase setup problems, or data inconsistency from incorrect uploads) the cluster should immediately terminate work. Note that the location of the HBase-Genomes jar file is in your s3 bucket, and the option –d must refer to the folder location where these files are stored (the “data” directory defined in the setup section above). Step 3 runs the actual mutation and stores the mutated fragments in HBase. If this step fails for any reason it should terminate the cluster as well. Steps 4 and 5 exports the database files back to your data directory in s3, and generates FASTA files from the mutated genome. These database files will include the mutated fragments created in the step above and will be written to a new directory under your data directory. If the FASTA file generation step is not added but the database files are exported, a cluster can be created later with two steps: Import and Generate.

When all steps have been specified click “Create cluster”. In the Cluster list you can view the job and check progress on individual steps. The cluster can be terminated at any point during the computation, however this will result in loss of any mutation data generated.

| \| 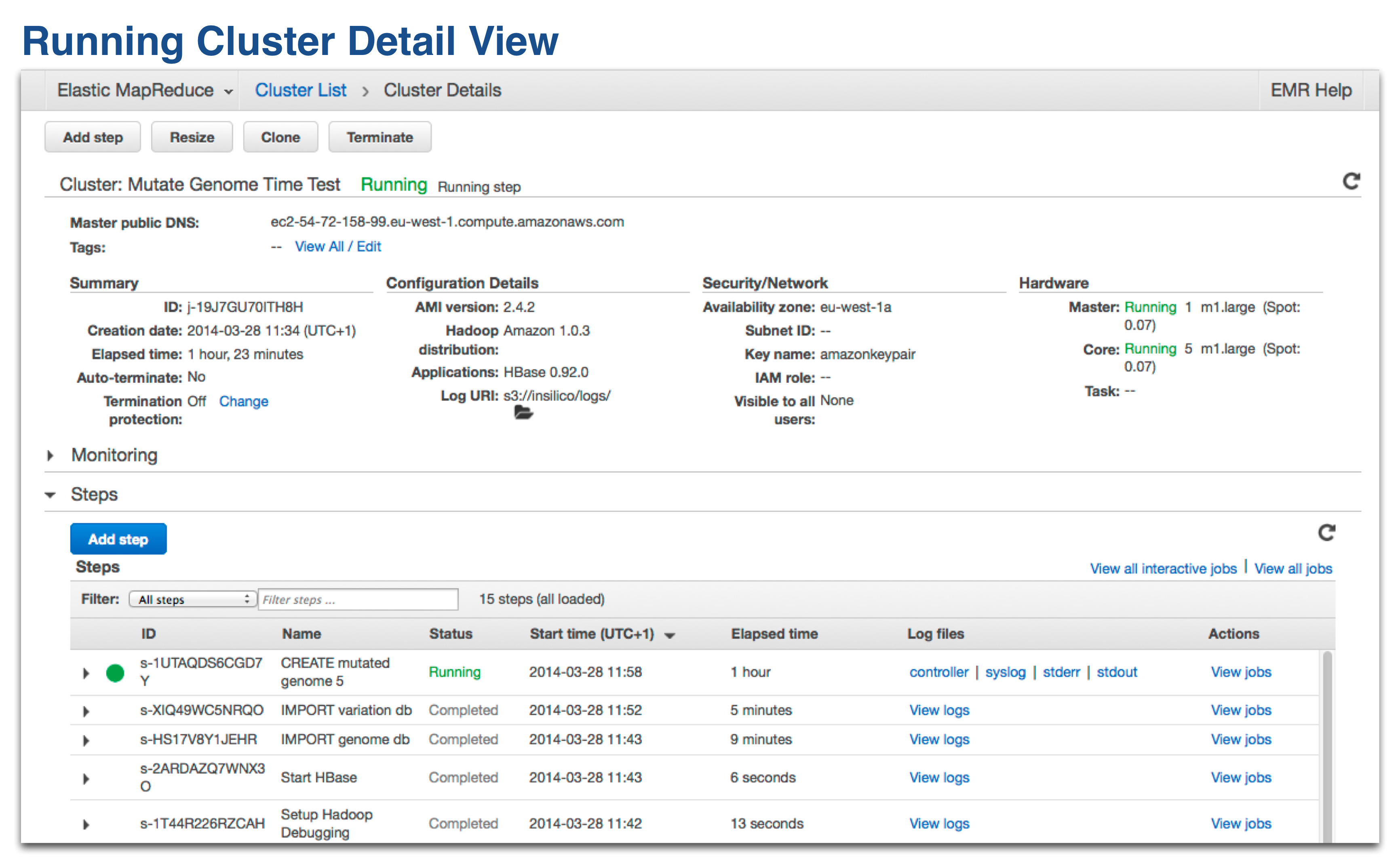 \| \| --- \| |
| --- | --- |
|  |

### Command-line elastic-mapreduce

A command-line Ruby tool is available for interacting with Elastic MapReduce clusters. To use this tool follow the installation instructions provided by AWS:

<https://docs.aws.amazon.com/ElasticMapReduce/latest/DeveloperGuide/emr-cli-reference.html>.

Download *run_mutate_aws.sh* from <http://insilicogenome.sourceforge.net>. Set the environment variable “EMR_HOME” to the directory containing the Ruby elastic-mapreduce script. The script requires three arguments: 1) A name for the new genome; 2) whether the cluster should terminate on failure (true/false); 3) the bucket you created on S3 that contains the jar file, hbase and logs folders; and 4) is optional, provide number of cores to use (default is 3).

sh run_mutate_aws.sh MyNewGenomeName true mybucket 10

This script assumes that the data directory created in S3 is called “hbase” and the log directory is “logs”. Additionally, these instructions assume that the cluster is being created using Spot Instances with a bid price of 0.07. The script can be edited to remove this if you want to use Reserved Instances.

All five steps described in the UI instructions are part of the shell script. After the script has run you can view the new cluster in the Web UI as shown above.
